# Spectral characterisation of ventricular intracardiac potentials in human post-ischaemic bipolar electrograms

**DOI:** 10.1038/s41598-022-08743-7

**Published:** 2022-03-21

**Authors:** Giulia Baldazzi, Marco Orrù, Giuliana Solinas, Mirko Matraxia, Graziana Viola, Danilo Pani

**Affiliations:** 1 Department of Informatics, Bioengineering, Robotics and Systems Engineering (DIBRIS), University of Genova, Genova, Italy; 2 Department of Electrical and Electronic Engineering (DIEE), University of Cagliari, Cagliari, Italy; 3 Department of Biomedical Sciences, University of Sassari, Sassari, Italy; 4 Medical Concept Lab, Sassari, Italy; 5 Division of Cardiology, San Francesco Hospital, Nuoro, Italy

**Keywords:** Biomedical engineering, Interventional cardiology

## Abstract

Abnormal ventricular potentials (AVPs) are frequently referred to as high-frequency deflections in intracardiac electrograms (EGMs). However, no scientific study performed a deep spectral characterisation of AVPs and physiological potentials in real bipolar intracardiac recordings across the entire frequency range imposed by their sampling frequency. In this work, the power contributions of post-ischaemic physiological potentials and AVPs, along with some spectral features, were evaluated in the frequency domain and then statistically compared to highlight specific spectral signatures for these signals. To this end, 450 bipolar EGMs from seven patients affected by post-ischaemic ventricular tachycardia were retrospectively annotated by an experienced cardiologist. Given the high variability of the morphologies observed, three different sub-classes of AVPs and two sub-categories of post-ischaemic physiological potentials were considered. All signals were acquired by the CARTO^®^ 3 system during substrate-guided catheter ablation procedures. Our findings indicated that the main frequency contributions of physiological and pathological post-ischaemic EGMs are found below 320 Hz. Statistical analyses showed that, when biases due to the signal amplitude influence are eliminated, not only physiological potentials show greater contributions below 20 Hz whereas AVPs demonstrate higher spectral contributions above ~ 40 Hz, but several finer differences may be observed between the different AVP types.

## Introduction

Ventricular tachycardia (VT) is an arrhythmia of ventricular origin frequently preceding sudden cardiac death; as such, this disorder is associated with increased mortality^1,2^. Implantable cardioverter–defibrillators can effectively terminate VT, but the shocks provided by these devices could increase mortality and affect patients’ quality of life without avoiding the arrhythmogenic onset^3,4^. Conversely, catheter ablation represents an effective therapeutic option for different forms of VT^5–9^. Recent research has demonstrated that substrate-guided mapping and catheter ablation during sinus rhythm may be a reasonable strategy to decrease arrhythmia recurrence^5,7^. Scar-related re-entrant circuits commonly constitute sustained monomorphic VT arrhythmia substrates^5,10^, which are caused by myocyte bundles surviving in incomplete scar areas, and, as such, give rise to slow-conduction paths and disordered propagation^11–14^. Whilst many different strategies and tools have been proposed in the literature^10,15–22^, substrate-guided mapping and ablation procedures generally target slow-conduction isthmuses, which are mainly identified by electrograms (EGMs) affected by abnormal ventricular potentials (AVPs)^23–26^, with the final aim of silencing them^16,17,27,28^. However, despite the abundance of technological and scientific advancements in the field, a deeper understanding of the re-entrant substrate remains necessary to optimise these clinical approaches^29^. Moreover, many authors have proposed different definitions of AVPs according to their temporal and morphological characteristics^5,22,27,30–35^, which, unfortunately, have been proven to be influenced by the infarct age in post-infarction VT^24^. AVPs are often qualitatively referred to as high-frequency deflections in intracardiac EGMs^21,22,27,34^, and different automated recognition algorithms are based on this assumption^36–38^. However, to the best of our knowledge, no spectral characterisation of AVPs and post-ischaemic physiological potentials from intracardiac bipolar recordings has been performed so far. In fact, spectral analysis and time–frequency analysis techniques have been previously exploited on ventricular EGMs only with the goal of identifying short-term frequency content variations induced by myocardial ischaemia^39^, distinguishing monomorphic and polymorphic VTs^40^, local and distant electrical activities^41^ and VT EGMs from normal sinus rhythm ones^42,43^, or targeting arrhythmogenic sites^36–38^, but not to identify peculiar signatures of AVPs in the frequency domain. A study^44^ investigating the effect of post-myocardial infarct on the frequency characteristics of ovine unipolar recordings acquired by multi-electrode plunge needles determined the relationship between high-frequency spectral characteristics and arrhythmogenic substrates. However, whilst the latter analysed peculiar histological and electrophysiological aspects, no evidence on AVPs was provided, nor their spectral contents were compared with post-ischaemic physiological EGM spectra.

In this work, a detailed spectral characterisation of AVPs and post-ischaemic physiological potentials in bipolar recordings is presented to provide deeper insights on these signals and move towards a comprehensive understanding of the arrhythmogenic substrate, which remains necessary in light of VT recurrence after ablation procedures^45^. The aim of this work is the identification of the spectral components exhibiting the highest informative and peculiar contribution for both AVPs and physiological potentials, as long as some peculiar features of the morphology of their spectra, in order to provide robust and hitherto unavailable characterization of these signals. Beyond providing novel insights on these signals, such a complete spectral characterization can be exploited as the basis for further studies aimed at their targeted automatic recognition. In this study, bipolar recordings were selected as they ease the detection of high-frequency components with good signal-to-noise ratio^46,47^. For this purpose, 450 real bipolar EGM segments acquired in sinus rhythm from seven patients affected by post-ischaemic VT were retrospectively analysed. Because of the high variability of their morphology, AVPs of three different types^31^ and post-ischaemic physiological potentials of two types^48^ were considered. On these signals, we investigated the power contributions by exploiting conventional spectral analysis methods combined with a sub-band partitioning. In particular, the periodogram power spectrum and power spectral density estimate (PSD) were selected as the most suitable tools to provide an easily understandable spectral characterization. Along with the absolute powers, the relative power contents on each sub-band and different spectral features were appraised to provide a complete overview. Compared to other published works, the significant novelty of our study is the detailed assessment of bipolar signals, which are typically inspected by the electrophysiologists during the ablation procedures, to identify the spectral signatures of AVPs and physiological potentials, along with their distinctive spectral characteristics, providing new insights also on their PSD morphology.

## Materials and methods

This retrospective study was based on a dataset consisting of 450 bipolar EGM segments acquired from seven patients (86% male; mean age, 64 ± 9 years), affected by post-ischaemic VT, at the San Francesco Hospital (Nuoro, Italy) between 2017 and 2018. This study on anonymised patient data was approved by the Independent ATS Ethical Committee (Azienda Tutela Salute, Sardegna) and performed following the principles outlined in the 1975 Helsinki Declaration, as revised in 2000. All patients provided their informed consent. Recordings were performed in sinus rhythm during left ventricular electroanatomic mapping by using the CARTO^®^ 3 system (Biosense Webster, Inc., Diamond Bar, CA, USA) at a sampling frequency of 1 kHz. After the procedure, radiofrequency catheter ablation followed the usual clinical protocols.

Bipolar intracardiac EGMs were recorded using PENTARAY™ (Biosense Webster, Inc.) 2-6-2 mm, by exploiting only the 2-mm spaced electrode pairs, THERMOCOOL SMARTTOUCH^®^ and THERMOCOOL SMARTTOUCH^®^ SF (Biosense Webster, Inc.) catheters. All signals were band-pass filtered between 16 and 500 Hz by the CARTO^®^ 3 acquisition system. Whilst the duration of each exported CARTO^®^ recording was 2.5 s, only the biopotential around the reference annotation was guaranteed to be acquired by the multielectrode catheter in effective contact with the endocardium. Therefore, a window of 200 ms before and 300 ms after the reference point was identified as the portion of interest for all subsequent analyses. According to the sampling frequency adopted in the CARTO^®^ 3 system (i.e. 1000 Hz) and the chosen duration of the analysis window for each EGM (500 ms), the available frequency resolution for the discrete-time frequency analyses was 2 Hz.

All intracardiac EGMs were manually labelled by an experienced cardiologist using an ad hoc MATLAB graphical user interface implemented for this purpose. Specifically, by exploiting the corresponding simultaneous surface ECG leads, we considered all abnormal potentials occurring after or during the corresponding QRS complexes, as in other works^31,49,50^, and all physiological potentials from post-ischaemic damaged substrates, as detailed hereafter.

***Abnormal potentials***, i.e. AVPs, were divided into three types^31^, to take into account the high variability of the morphologies of all the bipolar intracardiac EGMs:

- LP1: endocardial bipolar abnormal deflections spreading after the end of the corresponding surface QRS depolarisation,
- LP2: endocardial bipolar depolarisations starting during the corresponding surface QRS depolarisation but vanishing after its end, and
- EP: endocardial early EGM deflections completely falling within the corresponding surface QRS depolarisation boundaries.

***Physiological biopotentials*** (i.e., those without any abnormal deflection) originating from damaged myocardial substrates were also considered, dividing them into border-zone and scar-related types. Only these physiological potentials were categorised according to their peak-to-peak amplitudes (A_pp_) recorded in the bipolar EGMs. Conventionally^48^, endocardial border-zone potentials satisfied the constraint 0.5 mV < A_pp_ < 1.5 mV, whereas scar-related ones were characterised by A_pp_ < 0.5 mV. Amplitude categorisation was not applied to AVPs signals. All noisy or doubtful traces were discarded.

**Figure 1:**
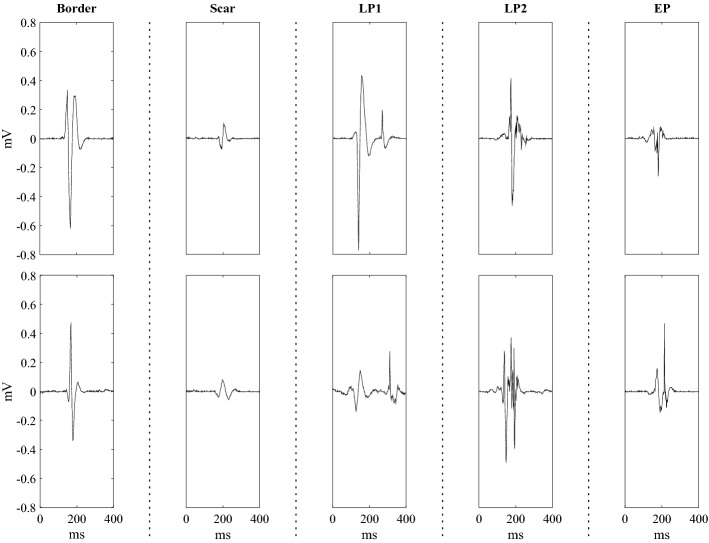
Prototypical examples for each EGM type. Two examples are represented on top and bottom rows for each EGM type. Specifically, from left to right, border-zone potentials, scar potentials, LP1 EGMs, LP2 EGMs and EP EGMs are depicted.

Over all the procedures, equal numbers of examples were randomly selected for each type of potential, (90 per type) to provide an effective and balanced comparative assessment. Figure 1 reports some typical examples of each EGM type.

The power contents for all bipolar EGMs were evaluated in the frequency domain. This analysis was carried out by adopting PSD and multiple spectral features to characterise the signals and their spectra better, as detailed in the following section.

### Spectral analysis methods

The spectral analysis included several steps. Firstly, the main frequency range of interest for these signals was identified, as described in "Definition of the main frequency range of interest", to reduce the dimensionality of the problem by focusing on the spectral range that contains the largest part of the signal power. We then assessed and compared the power contributions in different sub-bands to perform a fine-grained analysis of the EGM spectral content. Sub-band partitioning with a bandwidth of 20 Hz was performed, as also described in a previous study^44^, on the basis of the frequency resolution imposed by the chosen window length. This choice allowed reducing the impact of both noise and intrinsic limitations of the signal analysis methods adopted, by trading off robustness and accuracy of the characterisation.

The PSD method was exploited for spectral power estimation; here, the results were studied over sub-band partitions of the main frequency range of interest. Furthermore, we computed the absolute and relative power contents of the potentials in the different sub-bands because relative power analysis allows the suppression of the signal amplitude influence, mainly due to the far field component, which clearly affects the power estimated from the spectral analysis. Finally, different spectral features were evaluated to characterise the different PSD morphologies and contents of the EGM types.

#### Definition of the main frequency range of interest

We performed a preliminary identification of the main frequency range of interest in order to focus the signal frequency analysis on the components that provide the largest part of information, leading to a more effective and understandable sub-band analysis of ventricular endocardial potentials. This approach allowed us to restrict the number of sub-bands whilst still guaranteeing fine granularity during spectrum partitioning. To this end, for each EGM, we computed the PSD and estimated its total power as the area under the PSD curve over the entire frequency band limited by the Nyquist frequency. Then, we identified the frequency *f*_*H*_ corresponding to the value for which the 95% of the total PSD power was retrieved. Finally, considering the *f*_*H*_ values for each of the five EGM types, we computed the 95th percentiles (ξ_95_) separately for each EGM type and identified the lowest sub-band including the maximum ξ_95_. The frequency range of interest was defined as the band between zero and the upper boundary of that sub-band.

#### Spectral analysis by PSD

The power spectrum of the discrete-time signal represents its power as a function of the frequency bin ${\omega }_{k}$, which is the normalized frequency, defined as $\pi f/\left({f}_{s}/2\right)$, where ${f}_{s}$ represents the adopted sampling frequency. When the discrete Fourier transform (DFT) is used, ${\omega }_{k}$ assumes the discrete values $2\pi k/N$. In fact, in its simplest non-parametric estimate, the power spectrum can be computed from the DFT of the windowed signal of interest (${\varvec{x}}[n]$), consisting of $N$ samples, by normalising its squared magnitude as follows^51^:

1 $${\varvec{P}}{\varvec{S}}\left[{\omega }_{k}\right]= \frac{{\left|{\varvec{D}}{\varvec{F}}{\varvec{T}}\{{\varvec{x}}[n]\}\right|}^{2}}{{{S}_{1}}^{2}}$$

where:

2 $${\varvec{D}}{\varvec{F}}{\varvec{T}}\{{\varvec{x}}[n]\} = {\sum }_{n = 0}^{N-1}x[n] {e}^{-j2\pi kn/N}$$

and where ${S}_{1}$ is obtained by summing the values of the chosen window, while $n$ and $k$ span between 0 and $N-1$, being $N$ the signal length. Specifically, in this work, fast Fourier transform was adopted along with a rectangular window function. The latter was chosen to avoid attenuation of the signal at its borders, as border effects are still limited by the high-pass pre-processing stage of the recording system. In this application, the choice of the periodogram estimate was imposed by the short duration of the EGM, thus providing a single epoch for each intracardiac potential.

The spectral power was estimated as the area under the PSD curve. PSD can be computed by dividing the power spectrum by the effective noise equivalent bandwidth^51^. According to this assumption, PSD can be mathematically expressed as:

3 $${\varvec{P}}{\varvec{S}}{\varvec{D}}\left[{\omega }_{k}\right]= \frac{{\left|{\varvec{D}}{\varvec{F}}{\varvec{T}}\{{\varvec{x}}[n]\}\right|}^{2}}{{f}_{s} {S}_{2}}$$

where ${S}_{2}$ is defined as the sum of the squared window values. In this work, absolute power analysis was carried out by computing the area under the PSD curves in each sub-band.

The relative powers were also computed to avoid any influence in the analysis due to the amplitude of the signals, which is mainly ascribable to the far field component. These powers were estimated as the percentage ratio between the absolute power values of each sub-band and the area under the whole PSD curve in the main frequency range, similarly to a previous work^39^.

Moreover, a deeper characterization of the different PSD morphologies was obtained by exploiting some frequency-domain features^52^. Specifically, for each EGM, we considered:

- the mean frequency (MF), which is computed as the weighted sum of the power spectrum contents ${PS}_{j}$ and the corresponding frequencies ${f}_{j}$ with respect to the total power estimated in the main frequency range of interest ($P$):
  4 $$MF= \frac{{\sum }_{j}{(f}_{j} {PS}_{j})}{P}$$
  where $P= {\sum }_{j}{PS}_{j}$; on this basis, MF explains if the power spectrum contents are mostly localized at the higher or lower frequencies;
- the mean spectral power (MP), which is defined as the mean power in the main frequency range of interest and, as such, expresses how much power, on average, is contained under each PSD in that range;
- the maximum or peak frequency (PKF), which corresponds to the frequency at which the maximum of the power spectrum occurs;
- the power spectrum ratio (PSR), which is estimated as the ratio between the spectral power included near the PKF and the total power estimated in the main frequency range of interest $\left(P\right)$:
  5 $$PSR \left[\%\right]= 100\bullet \frac{{P}_{0}}{P}$$

where ${P}_{0}= {\sum }_{j=PKF-\delta }^{PKF+\delta }{P}_{j}$. Specifically, $\delta$ was set to be equal to 4 Hz. As such, the estimation was performed on a spectral interval equal to 8 Hz centred around the PKF to give an overview of how much relative power is concentrated around the PKF, highlighting how sharp the PSD morphology is around its maximum.

All computations were performed with MATLAB v2019b (MathWorks Inc., MA, USA).

#### Statistical analysis

The normality of all data distributions was preliminary assessed by Shapiro–Wilk’s test, and the homogeneity of variances was evaluated with Levene’s test. Data deviating from normality were presented as median values, and differences between groups were compared by the Kruskal–Wallis test. When the p value from Kruskal–Wallis test was statistically significant, a post hoc Conover’s non-parametric multiple comparison test^53,54^ was used to determine which group differed from the others. Several statistical inferences were simultaneously applied for all multiple comparisons, and the Bonferroni procedure for family-wise error rates was applied to control type-I errors by multiplying the uncorrected p-value with the total number of pair-wise tests; the adjusted p-value for all computations was reported. Following Bonferroni adjustment, p value < 0.025 was accepted as significant.

All statistical analyses were performed using STATA 16 (StataCorp LP, College Station, TX, USA).

## Results

### Identification of the main frequency range of interest

**Figure 2:**
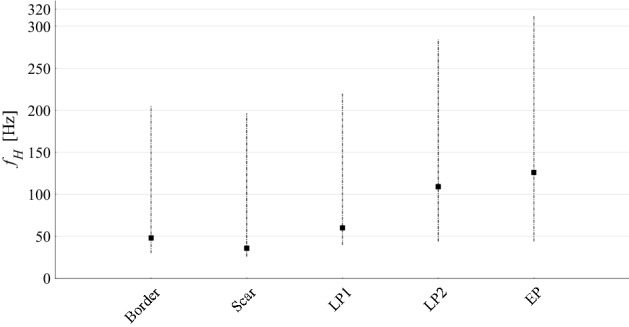
Distribution of the frequency values *f*_*H*_ including 95% of the total PSD power for each EGM type. Distributions of frequency limits *f*_*H*_ are reported as medians (squares) and 5th and 95th percentiles.

The distributions of the *f*_*H*_ values for the five different EGM types are presented in Fig. 2 as median and 5th and 95th percentile values. Visual inspection indicates that the spectral power analysis can be effectively carried out on all contents below approximately 320 Hz, which includes all of the most extreme *f*_*H*_ values.

### Spectral investigations

**Figure 3:**
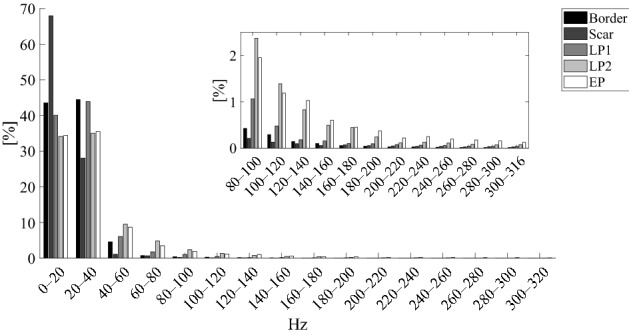
Median relative power contents in the different sub-bands for each EGM type. Median values of relative power contents obtained in the different spectral ranges are reported for Border (black bars), Scar (darker grey bars), LP1 (middle grey bars), LP2 (lighter grey bars) and EP (white bars) EGMs. A zoom on the upper sub-bands (i.e., from 80 to 320 Hz) is also provided to allow for a better visualization of the relative power contributions at the higher frequencies. The legend graphically details the association between colours and the different EGM types.

**Figure 4:**
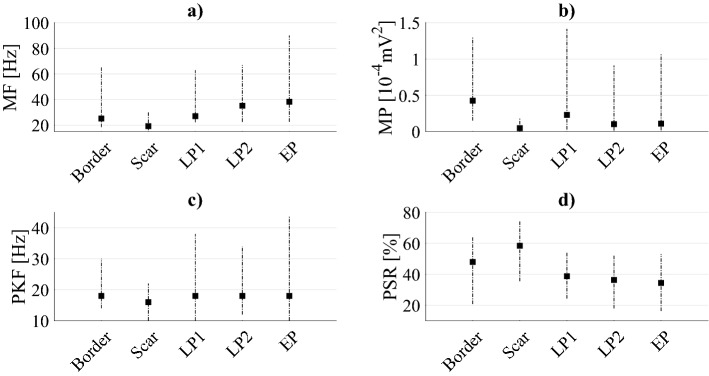
Spectral features estimated for all EGM types. Spectral feature results are reported as medians (black squares) and 5th and 95th percentiles. Specifically, in (**a**) the distributions of mean frequency (MF) values are represented for all EGM types, whereas in (**b**–**d**) those referred to the mean spectral power (MP), the maximum or peak frequency (PKF) and the power spectrum ratio (PSR), respectively.

In this section, all spectral power estimations are reported as pair-wise comparisons amongst the different EGMs types. The results are organised into three groups according to the nature of the EGMs included in the comparison. In the first group, all the considered post-ischaemic physiological potentials (border and scar) were studied. In the second group, all AVPs (LP1, LP2 and EP) were examined; in the third group, physiological potentials were compared with AVPs. Table 1 summarises the spectral power results of pair-wise comparisons for different spectral ranges. In the table, the different groups are identified by vertical dashed lines. The absolute and relative power contents obtained for each EGM type in the different spectral ranges are reported in Supplementary Tables S1 and S2 online. Figure 3 presents the results of relative power analysis in terms of median values graphically, and Fig. 4 describes the results in terms of spectral features.

**Table 1:**
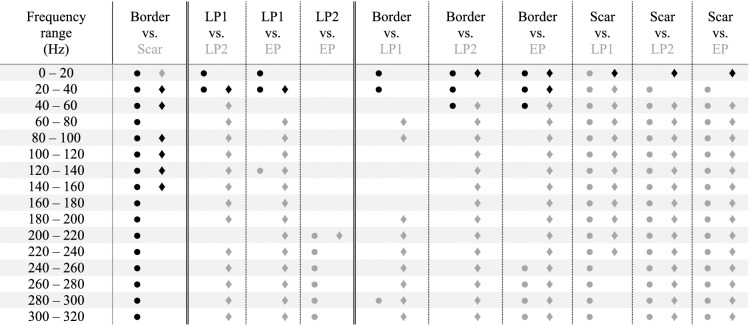
Statistically significant differences (p < 0.025) amongst the different pair-wise comparisons obtained via PSD analysis considering absolute (●) and relative (♦) power contents in the different spectral sub-bands by the post hoc Conover’s non-parametric multiple comparison statistical test.

Wherever statistically significant differences could not be found, no symbol was reported. The colour of each symbol (black or grey) is associated with the EGM type, indicated in the heading line of that column with the same colour, exhibiting the highest median power content in that specific comparison (e.g., in the first column, which is related to the comparison between Border and Scar, black symbols are reported whenever an higher contribution of the border EGMs was detected, whereas grey symbols are present in case of significantly greater contribution of scar EGMs).

#### Post-ischaemic physiological potentials

Conversely from absolute power and MP results, which were driven by the signal amplitude and confirmed that border power contents were significantly higher than scar ones in all examined ranges (p < 0.0001), relative power analyses revealed how scar power values were significantly higher than border ones at the lowest frequencies, i.e. 0–20 Hz (p < 0.0001). However, border relative powers were again higher than scar ones only up to 160 Hz, specifically in 20–60 Hz (p < 0.0001) and 80–160 Hz (p < 0.0074), although statistical significance was nearly achieved in the 60–80 Hz range (p = 0.0391). Scar MF and PKF values were significantly lower (p < 0.0001 for both cases) whereas scar PSR estimations were significantly higher (p < 0.0001) than border ones, thus confirming that this EGM type demonstrates its main contribution at lower frequencies than border cases.

#### Post-ischaemic abnormal potentials

##### LP1 and LP2 potentials

In terms of MP, our experiments revealed significantly higher values of LP1 than LP2 (p < 0.0001). Although absolute power analyses showed statistically significant differences only in the 0–40 Hz range, in which LP1 values were significantly higher than LP2 ones (p < 0.0001), relative power analysis revealed a different scenario. In this case, LP1 contents exceeded LP2 ones at low frequencies, in particular in the 20–40 Hz range (p = 0.0007), whereas LP2 showed significantly higher contributions at all the higher frequencies (p < 0.0120), except in the 200–220 Hz sub-band, in which no statistical significance was achieved. This finding was confirmed by the MF analysis, in which LP1 showed lower mean frequencies than LP2 (p = 0.0001). No statistical evidence was found between PKF and PSR.

##### LP1 and EP potentials

This comparison is similar to the previous one. MP values were significantly higher for LP1 than EP (p < 0.0001). Although absolute power analysis showed statistically significant significances mainly in the 0–40 Hz range, in which LP1 contributions were considerably higher than EP ones (p < 0.0001), relative power analysis demonstrated several statistically significant differences between 20 and 320 Hz. Specifically, although LP1 showed higher median contents at low frequencies, i.e. 20–40 Hz (p = 0.0002), EP values consistently exceeded those of LP1 between 60 and 320 Hz (p < 0.0249). Significant lower-frequency contributions for LP1 with respect to EP were also found when MF values were assessed (p < 0.0001).

##### LP2 and EP potentials

In general, no significant difference was found between the LP2 and EP classes below 200 Hz. For higher frequencies, EP demonstrated significantly higher values in the 200–320 Hz range (p < 0.0233) during absolute power analysis but only in the 200–220 Hz range in the corresponding relative power analysis (p = 0.0187).

#### Physiological versus abnormal potentials

##### Border and LP1 potentials

The MPs of border EGMs were significantly higher than those of LP1 EGMs (p = 0.0002). Statistical differences were rarely observed between these EGM types when absolute powers were analysed, i.e. essentially in the 0–40 Hz (p < 0.0217) range, in which border powers exceeded LP1 ones. However, when relative powers were analysed, LP1 spectral contents exceeded border ones in the range of 60–100 Hz (p < 0.0091) and above 180 Hz (p < 0.0156). Furthermore, border PSR values were significantly higher than LP1 ones (p = 0.0017), thus suggesting a lower dispersion of power contributions around the maximum frequency in border EGMs.

##### Border and LP2 potentials

Border EGMs showed statistically higher MP values than LP2 (p < 0.0001). During absolute spectral analysis, statistically significant differences were noted only at frequencies below 60 Hz, where border power contents exceeded LP2 ones (p < 0.0070). However, LP2 and border EGMs significantly differed in terms of relative power in all the sub-bands except in the 20–40 Hz one. Table 1 reveals that LP2 showed higher power contents in higher frequency ranges, i.e. between 40 and 320 Hz (p < 0.0001); the opposite behaviour was observed at the lowest frequencies, i.e. 0–20 Hz (p = 0.0048), where border EGMs exhibited higher power contributions. The main low-frequency contributions of border EGMs are also reflected by the MF results, in which border contributions were significantly lower than LP2 ones (p < 0.0001). PSR values suggested a major power gathering around the PKF for border EGMs (p < 0.0001).

##### Border and EP potentials

The MP values of border EGMs were significantly higher than those of EP EGMs (p < 0.0001). Conversely, EP MF values significantly exceeded border ones (p < 0.0001) with lower PSR contents (p < 0.0001). Absolute power analysis revealed statistically significant differences below 60 Hz, in which border power contents exceeded EP ones (p < 0.0156), and above 240 Hz, where the opposite trend was noted (p < 0.0063). In terms of relative power, border and EP EGMs significantly differed in all the sub-bands, with border potentials showing higher contributions at lower frequencies (i.e. 0–40 Hz, p < 0.0197) and EP above 40 Hz (p < 0.0001).

##### Scar and LP1 potentials

Scar EGMs consistently showed lower contributions than LP1 when absolute powers, MP, MF and PKF were examined (p < 0.0001). However, scar relative power values exceeded LP1 one in the lowest sub-band, i.e. 0–20 Hz (p < 0.0001, see Table 1). LP1 power contents were significantly higher than scar ones at higher frequencies, i.e. 20–240 Hz (p < 0.0119). Furthermore, scar PSRs exceeded LP1 ones (p < 0.0001). These results clearly indicate that scar EGMs exhibited lower powers in absolute terms, but higher relative low-frequency contents (see Fig. 3) with a considerable amount of spectral power around their PKFs, which are typically observed below 25 Hz, as can be seen in Fig. 4.

##### Scar and LP2 potentials

Scar EGMs showed significantly lower power contributions than LP2 in terms of MP (p < 0.0001) and in the absolute power analysis at frequencies above 20 Hz (p < 0.0001). However, similarly to the previous case, the relative power contributions of these potentials demonstrated some interesting characteristics. Specifically, scar EGMs showed higher relative power contents at the lowest frequencies, i.e. below 20 Hz (p < 0.0001), but lower contents at higher frequencies, i.e. above 40 Hz (p < 0.0001). As in the previous case, this finding was confirmed by the trends of MF and PKF, in which scar contents were significantly lower than LP2 ones (p < 0.0001), and PSR values, in which scar EGMs exceeded LP2 ones (p < 0.0001).

##### Scar and EP potentials

Scar EGMs showed significantly lower powers than EP EGMs in terms of MP (p < 0.0001). The comparison in terms of absolute and relative power analysis led to the same results obtained in the previous case. The MF and PKF distributions confirmed the previous findings, thus suggesting higher scar contributions than EP ones (p < 0.0001 and p = 0.0002, respectively) at low frequencies, with major gathering around PKF in terms of PSR rather than EP (p < 0.0001).

## Discussion

In this work, different spectral power analyses were performed to characterise abnormal and physiological post-ischaemic bipolar potentials and provide an accurate and quantitative comparative assessment amongst the different EGM types analysed. The results were grouped in terms of comparisons between physiological potentials, abnormal potentials and physiological versus abnormal potentials, i.e. AVPs.

Although the absolute power analysis is influenced by the amplitude of the signals, which is mainly driven by the far field component, its results are quite interesting when compared to the relative power analysis and the insights synthetically provided by the identified spectral features. According to the absolute power analysis results (see Table 1), scar EGMs consistently demonstrated lower power values than all other types of potentials, regardless of the frequency range analysed. However, the spectral feature analysis results (see Fig. 4) clearly show that scar MF and PKF are typically below 30 Hz, around which substantial power contributions are concentrated, as indicated by their higher PSRs. These findings suggest that scar power spectra are quite condensed around their maximum and may confirm the association between the lowest-frequency contributions and the scar areas, as further demonstrated by the relative power analysis results. While PKF allowed to statistically emphasize scar potentials, PSR contents allowed to discriminate physiological potentials from AVPs, since both border and scar EGMs showed major power contributions around PKF (i.e., higher PSR in Fig. 4). MF analysis seemed to delineate three clusters, namely, border and LP1, LP2 and EP, and scar.

However, the absolute power results in Table 1 suggest that, below 40 Hz, border EGMs show higher contribution than all AVPs and that, amongst AVPs, LP1 EGMs involve greater slow components. The same finding could be confirmed by looking at the lower MF values in Fig. 4 for LP1 and border EGMs. Conversely, EPs showed higher contributions than border EGMs above 240 Hz, revealing significant high-frequency components, which also emerged in the comparison with LP2 above 200 Hz. This finding was supported by the MF evaluation, in which the values of EP were found to be generally higher than those of all other EGM types.

Although absolute power analysis results are interesting for the spectral characterization of physiological potentials and AVPs, the relative power analysis (see Table 1) provides several novel insights into the characterisation of these signals. Overall, our results indicate that physiological EGMs have greater contributions at lower frequencies (i.e. mainly below 20 Hz) than pathological ones, as conceivable according to the fragmented nature of AVPs. In particular, scar-related EGMs consistently indicated higher contributions at lower frequencies (i.e. 0–20 Hz) than all the other physiological and pathological types, which, however, gradually decreased when moving towards higher sub-bands. These findings are at least partially in accordance with previous scientific studies on post-ischaemic epicardial EGMs^39,40^. In fact, Mor-Avi and Akselrod^39^ demonstrated that myocardial ischaemia is responsible for a shift of the major frequency contents below 40 Hz whilst attenuating high-frequency spectral components. Sierra et al.^40^ concluded that most of the signal energy is concentrated below 30 Hz in monomorphic and polymorphic VT recordings. Relative powers of LP2 and EP exhibited significantly greater values from 40 Hz than post-ischaemic physiological potentials, even though statistical significance was not achieved on some spectral ranges (see Table 1). Conversely, whilst LP1 EGMs showed significantly higher power contents than scar potentials in the 20–240 Hz range, they generally did not assume significantly higher values than border potentials until 180 Hz. As such, AVPs presented greater relative powers at higher frequencies with respect to physiological potentials, with some differences in spectral ranges depending on the AVP type. Moreover, as regards the significances amongst AVPs, while LP1 showed lower power contributions than LP2 and EP mainly above 60 Hz, LP2 and EP EGMs statistically differed in 200–220 Hz sub-band.

**Figure 5:**
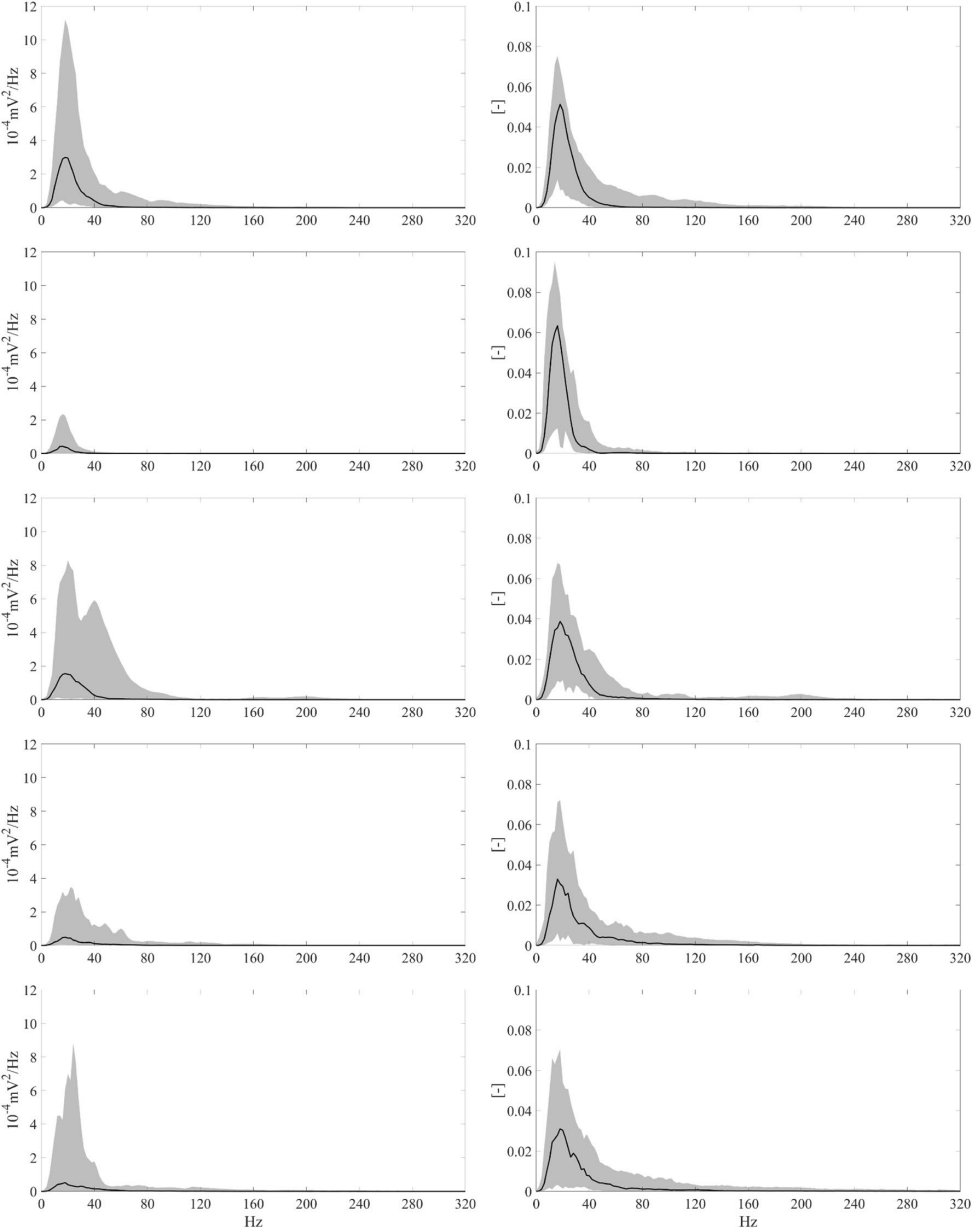
Absolute and normalised PSDs for all EGM types. Representative PSDs for both physiological and pathological signals in terms of medians (black solid line) and 5th and 95th percentiles (grey zones). From top to bottom: PSDs for border, scar, LP1, LP2 and EP EGMs. The left column illustrates standard PSD curves, whilst the right column depicts the same PSD curves after normalisation so that the area under each curve is unitary, thus allowing for a comparative overview of both absolute and relative power contents for all EGM types in different sub-bands.

In summary, AVPs demonstrated higher relative power contributions at higher frequencies (i.e. mainly above 40 Hz) when compared with physiological potentials, as can be also deduced from Fig. 5. These findings are quite consistent with the a priori assumptions described in one work^36^, in which spectral contents above 80 Hz were identified as a marker of highly fragmented EGMs, and another study^37^, which identified the range of 70–180 Hz to be the useful spectral range for the arrhythmogenic potentials identification. Moreover, our results partially agree with another study^38^, which indicated the 40–100 Hz range to be of interest for fractionated EGM recognition, and an earlier analysis^44^, in which arrhythmogenic scar potentials showed significantly higher root mean square powers than non-arrhythmogenic ones from approximately 40 Hz, at least in dense scar tissue. However, in all these previous studies, no justification behind the choice of the specific spectral range was provided. Indeed, differently to the previous works, our study characterises the spectral signatures of human endocardial post-ischaemic EGMs, both normal and abnormal, quantitatively by analysing their power contents in different sub-bands and looking at their spectral morphologies. Our analysis not only focuses on scar-related and arrhythmogenic substrates but also describes, in detail, the different AVP and physiological EGM types, thus providing additional important information for their characterisation.

Whilst the rigorous assessment described above allows for studies on statistical differences, the generalisability of our results is affected by some limitations of this study. Specifically, our analysis was carried out on a limited set of bipolar EGMs acquired from seven post-ischaemic VT patients. Thus, larger datasets are necessary to reinforce the results or reduce the strength of our findings. Moreover, all recordings were annotated by a single cardiologist, whose experience may have biased the subsequent analyses. Furthermore, the labelling process and the subsequent analyses were carried out simply based on the signals as they were recorded by the CARTO^®^ 3 system and as exploited during the clinical procedures, without taking into account any dependence from wavefront directionality and electrode parameters, which however affect the substrate-guided mapping techniques inherently^55–58^.

Other studies are in progress to address the far-field effect on AVP power estimations to provide a deeper characterisation of these potentials after the influence of global ventricular depolarisation is removed.

## Conclusions

Despite the widespread association of AVPs with high-frequency spectral contents in post-ischaemic intracardiac bipolar EGMs, a rigorous spectral analysis of these signals was missing in the scientific literature. In this work, we addressed this gap and reveal that the main frequency contributions of physiological and pathological post-ischaemic EGMs are found below 320 Hz. Moreover, when amplitude influences are eliminated, physiological potentials show greater contributions at lower frequencies whereas AVPs demonstrate higher spectral contributions at frequencies above ~ 40 Hz. Conversely, when looking at AVPs only, LP1 showed lower power contributions than LP2 and EP mainly above 60 Hz, whereas LP2 and EP EGMs statistically differed in 200–220 Hz sub-band. In the light of the above-mentioned spectral signatures of AVPs and physiological potentials, our findings may be valuable not only because they represent a spectral characterisation of these potentials, moving a step forward in the direction of a deeper understanding of arrhythmogenic substrate mechanisms, but also to support the development of automatic tools for the efficient and targeted distinction of different physiological and abnormal EGM types.

## Supplementary Information

Supplementary Information.

## References

[1] Benjamin EJ (2019). Heart disease and stroke statistics—2019 update: A report from the American Heart Association. Circulation.

[2] Koplan BA, Stevenson WG (2009). Ventricular tachycardia and sudden cardiac death. Mayo Clin. Proc..

[3] Harris P, Lysitsas D (2015). Ventricular arrhythmias and sudden cardiac death. BJA Educ..

[4] Wissner E, Stevenson WG, Kuck K-H (2012). Catheter ablation of ventricular tachycardia in ischaemic and non-ischaemic cardiomyopathy: Where are we today? A clinical review. Eur. Heart J..

[5] Cronin EM (2019). 2019 HRS/EHRA/APHRS/LAHRS expert consensus statement on catheter ablation of ventricular arrhythmias. Hear. Rhythm.

[6] Martinez BK (2020). Systematic review and meta-analysis of catheter ablation of ventricular tachycardia in ischemic heart disease. Hear. Rhythm.

[7] Dukkipati SR (2017). Catheter ablation of ventricular tachycardia in structural heart disease: Indications, strategies, and outcomes—Part II. J. Am. Coll. Cardiol..

[8] Tung R (2015). Freedom from recurrent ventricular tachycardia after catheter ablation is associated with improved survival in patients with structural heart disease: An International VT Ablation Center Collaborative Group study. Hear. Rhythm.

[9] Sapp JL (2016). Ventricular tachycardia ablation versus escalation of antiarrhythmic drugs. N. Engl. J. Med..

[10] Natale A (2010). Venice Chart International Consensus document on ventricular tachycardia/ventricular fibrillation ablation. J. Cardiovasc. Electrophysiol..

[11] de Bakker JMT (1988). Reentry as a cause of ventricular tachycardia in patients with chronic ischemic heart disease: Electrophysiologic and anatomic correlation. Circulation.

[12] de Bakker JMT (1993). Slow conduction in the infarcted human heart. ‘Zigzag’ course of activation. Circulation.

[13] Stevenson WG (1997). Exploring postinfarction reentrant ventricular tachycardia with entrainment mapping. J. Am. Coll. Cardiol..

[14] Gardner PI, Ursell PC, Fenoglio JJJ, Wit AL (1985). Electrophysiologic and anatomic basis for fractionated electrograms recorded from healed myocardial infarcts. Circulation.

[15] Briceño DF (2017). Substrate ablation of ventricular tachycardia: Late potentials, scar dechanneling, local abnormal ventricular activities, core isolation, and homogenization. Cardiac. Electrophysiol. Clin..

[16] Santangeli P, Frankel DS, Marchlinski FE (2014). End points for ablation of scar-related ventricular tachycardia. Circ. Arrhythm. Electrophysiol..

[17] Santangeli P, Marchlinski FE (2016). Substrate mapping for unstable ventricular tachycardia. Hear. Rhythm.

[18] Komatsu Y (2014). Substrate-based approach for ventricular tachycardia in structural heart disease: Tips for mapping and ablation. J. Arrhythmia.

[19] Kitamura T (2019). Substrate mapping and ablation for ventricular tachycardia in patients with structural heart disease: How to identify ventricular tachycardia substrate. J. Innov. Card. Rhythm Manage..

[20] Bourier F (2019). Is it feasible to offer ‘targeted ablation’ of ventricular tachycardia circuits with better understanding of isthmus anatomy and conduction characteristics?. EP Eur..

[21] Aziz Z, Tung R (2018). Novel mapping strategies for ventricular tachycardia ablation. Curr. Treat. Options Cardiovasc. Med..

[22] Huang SKS, Miller JM (2019). Catheter Ablation of Cardiac Arrhythmias.

[23] Klein H (1982). Intraoperative electrophysiologic mapping of the ventricles during sinus rhythm in patients with a previous myocardial infarction. Identification of the electrophysiologic substrate of ventricular arrhythmias. Circulation.

[24] Bogun F (2005). Electrogram characteristics in postinfarction ventricular tachycardia. J. Am. Coll. Cardiol..

[25] Hsia HH, Lin D, Sauer WH, Callans DJ, Marchlinski FE (2009). Relationship of late potentials to the ventricular tachycardia circuit defined by entrainment. J. Interv. Card. Electrophysiol. Int. J. Arrhythm. Pacing.

[26] Stevenson WG (1989). Fractionated endocardial electrograms are associated with slow conduction in humans: Evidence from pace-mapping. J. Am. Coll. Cardiol..

[27] Jaïs P (2012). Elimination of local abnormal ventricular activities: A new end point for substrate modification in patients with scar-related ventricular tachycardia. Circulation.

[28] Vergara P (2012). Late potentials abolition as an additional technique for reduction of arrhythmia recurrence in scar related ventricular tachycardia ablation. J. Cardiovasc. Electrophysiol..

[29] Graham AJ, Orini M, Lambiase PD (2017). Limitations and challenges in mapping ventricular tachycardia: New technologies and future directions. Arrhythmia Electrophysiol. Rev..

[30] Cassidy DM (1986). Endocardial catheter mapping in patients in sinus rhythm: Relationship to underlying heart disease and ventricular arrhythmias. Circulation.

[31] Silberbauer J (2014). Noninducibility and late potential abolition: A novel combined prognostic procedural end point for catheter ablation of postinfarction ventricular tachycardia. Circ. Arrhythmia Electrophysiol..

[32] Haqqani HM (2009). Fundamental differences in electrophysiologic and electroanatomic substrate between ischemic cardiomyopathy patients with and without clinical ventricular tachycardia. J. Am. Coll. Cardiol..

[33] Tsiachris D (2015). Electroanatomical voltage and morphology characteristics in postinfarction patients undergoing ventricular tachycardia ablation: Pragmatic approach favoring late potentials abolition. Circ. Arrhythm. Electrophysiol..

[34] Sacher F (2015). Substrate mapping and ablation for ventricular tachycardia: The LAVA approach. J. Cardiovasc. Electrophysiol..

[35] Nakahara S (2010). Characterization of the arrhythmogenic substrate in ischemic and nonischemic cardiomyopathy implications for catheter ablation of hemodynamically unstable ventricular tachycardia. J. Am. Coll. Cardiol..

[36] Campos B, Jauregui ME, Marchlinski FE, Dixit S, Gerstenfeld EP (2015). Use of a novel fragmentation map to identify the substrate for ventricular tachycardia in postinfarction cardiomyopathy. Hear. Rhythm.

[37] Lin C-Y (2016). Simultaneous amplitude frequency electrogram transformation (SAFE-T) mapping to identify ventricular tachycardia arrhythmogenic potentials in sinus rhythm. JACC Clin. Electrophysiol..

[38] Kuroki K (2018). New substrate-guided method of predicting slow conducting isthmuses of ventricular tachycardia: Preliminary analysis to the combined use of voltage limit adjustment and fast-fourier transform analysis. Circ. Arrhythmia Electrophysiol..

[39] Mor-Avi V, Akselrod S (1990). Spectral analysis of canine epicardial electrogram. Short-term variations in the frequency content induced by myocardial ischemia. Circ. Res..

[40] Sierra G (1997). Spectral analysis of electrograms during ventricular tachycardia in a canine model: Relation with epicardial isochronal maps. J. Electrocardiol..

[41] Cabo C, Wharton JM, Wolf PD, Ideker RE, Smith WM (1990). Activation in unipolar cardiac electrograms: A frequency analysis. IEEE Trans. Biomed. Eng..

[42] Pannizzo F, Furman S (1988). Frequency spectra of ventricular tachycardia and sinus rhythm in human intracardiac electrograms-application to tachycardia detection for cardiac pacemakers. IEEE Trans. Biomed. Eng..

[43] Minami K, Nakajima H, Toyoshima T (1999). Real-time discrimination of ventricular tachyarrhythmia with Fourier-transform neural network. IEEE Trans. Biomed. Eng..

[44] Morellato J (2018). Quantitative spectral assessment of intracardiac electrogram characteristics associated with post infarct fibrosis and ventricular tachycardia. PLoS ONE.

[45] Darma A (2020). Predictors of long-term mortality after catheter ablation of ventricular tachycardia in a contemporary cohort of patients with structural heart disease. Eur. Eur. Pacing Arrhythmias Card. Electrophysiol. J. Work. Groups Card. Pacing, Arrhythmias, Card. Cell. Electrophysiol. Eur. Soc. Cardiol.

[46] Goldberger J, Ng J (2010). Practical Signal and Image Processing in Clinical Cardiology.

[47] de Bakker JMT (2019). Electrogram recording and analyzing techniques to optimize selection of target sites for ablation of cardiac arrhythmias. Pacing Clin. Electrophysiol..

[48] Marchlinski FE, Callans JD, Gottlieb CD, Erica Z (2000). Linear ablation lesions for control of unmappable ventricular tachycardia in patients with ischemic and nonischemic cardiomyopathy. Circulation.

[49] Baldazzi G, Orrù M, Matraxia M, Viola G, Pani D (2019). Automatic recognition of ventricular abnormal potentials in intracardiac electrograms. Comput. Cardiol..

[50] Baldazzi, G., Orrù, M., Matraxia, M., Viola, G. & Pani, D. Supervised classification of ventricular abnormal potentials in intracardiac electrograms. In *2020 Computing in Cardiology* 1–4 (2020). 10.22489/CinC.2020.397.

[51] Heinzel G, Rüdiger A, Schilling R (2002). Spectrum and spectral density estimation by the Discrete Fourier transform (DFT), including a comprehensive list of window functions and some new flat-top windows. Max Plank Inst..

[52] Phinyomark, A., Thongpanja, S., Hu, H., Phukpattaranont, P. & Limsakul, C. The usefulness of mean and median frequencies in electromyography analysis. In *Computational Intelligence in Electromyography Analysis—A Perspective on Current Applications and Future Challenges* 195–220 (2012). 10.5772/50639.

[53] Conover WJ (1999). Practical Nonparametric Statistics.

[54] Conover, W. J. & Iman, R. L. On multiple-comparisons procedures. *Los Alamos Sci. Lab. Tech. Rep. LA-7677-MS* **1**, 14 (1979).

[55] Josephson ME, Anter E (2015). Substrate mapping for ventricular tachycardia: Assumptions and misconceptions. JACC Clin. Electrophysiol..

[56] Tung R, Josephson ME, Bradfield JS, Shivkumar K (2016). Directional influences of ventricular activation on myocardial scar characterization. Circ. Arrhythmia Electrophysiol..

[57] Beheshti M (2018). Determinants of atrial bipolar voltage: Inter electrode distance and wavefront angle. Comput. Biol. Med..

[58] Yamaguchi T, Fukui A, Node K (2019). Bipolar voltage mapping for the evaluation of atrial substrate: Can we overcome the challenge of directionality?. J. Atr. Fibrillation.

