# Supplementary material for: Spectral characterisation of ventricular intracardiac potentials in human post-ischaemic bipolar electrograms
Source: Sci Rep. 2022 Mar 21;12:4782. doi: 10.1038/s41598-022-08743-7 (PMC8938475; doi:10.1038/s41598-022-08743-7)
Supplement: Supplementary file 1 — Supplementary Information. [file 41598_2022_8743_MOESM1_ESM.docx]

# Spectral characterisation of ventricular intracardiac potentials in human post-ischaemic bipolar electrograms

Giulia Baldazzi^1,2,*^, Marco Orrù^2^, Giuliana Solinas^3^, Mirko Matraxia^4^, Graziana Viola^5^ and Danilo Pani^2^

*^1^ Department of Informatics, Bioengineering, Robotics and Systems Engineering (DIBRIS), University of Genova, Genova, Italy*

*^2^ Department of Electrical and Electronic Engineering (DIEE), University of Cagliari, Cagliari, Italy*

*^3^* *Department of Biomedical Sciences, University of Sassari, Sassari, Italy*

*^4^ Medical Concept Lab, Sassari, Italy*

*^5^ Division of Cardiology, San Francesco Hospital, Nuoro, Italy*

^*^corresponding author: giulia.baldazzi@unica.it

# Supplementary Material

Supplementary Table S1. **Absolute power contents obtained for all EGM types in the different spectral ranges.**

Values are reported in 10^−4^ mV^2^ in terms of medians (in bold) and 5^th^ and 95^th^ percentiles (in brackets).

| Frequency  range  (Hz) | Border | Scar | LP1 | LP2 | EP |
| --- | --- | --- | --- | --- | --- |
| 0 – 20 | **27.21**  [4.43 ; 97.92] | **4.06**  [0.27 ; 21.47] | **14.32**  [1.51 ; 66.99] | **4.68**  [0.97 ; 27.95] | **5.68**  [0.37 ; 62.20] |
| 20 – 40 | **23.98**  [5.84 ; 105.39] | **1.93**  [0.12 ; 10.18] | **17.73**  [1.94 ; 97.39] | **5.16**  [0.83 ; 48.35] | **6.43**  [0.55 ; 94.46] |
| 40 – 60 | **2.92**  [0.20 ; 25.13] | **0.05**  [0.01 ; 1.28] | **2.38**  [0.15 ; 81.73] | **1.71**  [0.15 ; 24.70] | **1.98**  [0.04 ; 10.36] |
| 60 – 80 | **0.67**  [0.03 ; 14.63] | **0.03**  [0.00 ; 0.45] | **0.63**  [0.07 ; 23.25] | **0.65**  [0.09 ; 6.30] | **0.62**  [0.06 ; 6.84] |
| 80 – 100 | **0.39**  [0.03 ; 7.71] | **0.01**  [0.00 ; 0.21] | **0.34**  [0.04 ; 7.89] | **0.32**  [0.03 ; 4.29] | **0.36**  [0.03 ; 4.40] |
| 100 – 120 | **0.19**  [0.01 ; 4.99] | **0.01**  [0.00 ; 0.16] | **0.18**  [0.01 ; 2.05] | **0.16**  [0.01 ; 3.90] | **0.21**  [0.01 ; 4.14] |
| 120 – 140 | **0.13**  [0.00 ; 3.00] | **0.00**  [0.00 ; 0.13] | **0.09**  [0.01 ; 0.92] | **0.10**  [0.01 ; 3.31] | **0.19**  [0.01 ; 3.62] |
| 140 – 160 | **0.10**  [0.01 ; 1.76] | **0.00**  [0.00 ; 0.10] | **0.06**  [0.01 ; 1.28] | **0.07**  [0.01 ; 2.02] | **0.07**  [0.00 ; 2.02] |
| 160 – 180 | **0.04**  [0.01 ; 1.36] | **0.00**  [0.00 ; 0.08] | **0.05**  [0.00 ; 2.68] | **0.07**  [0.01 ; 1.44] | **0.07**  [0.01 ; 1.51] |
| 180 – 200 | **0.03**  [0.00 ; 0.99] | **0.00**  [0.00 ; 0.03] | **0.04**  [0.01 ; 3.20] | **0.04**  [0.00 ; 0.90] | **0.06**  [0.00 ; 1.57] |
| 200 – 220 | **0.02**  [0.00 ; 0.99] | **0.00**  [0.00 ; 0.03] | **0.03**  [0.00 ; 2.86] | **0.01**  [0.00 ; 0.36] | **0.04**  [0.00 ; 1.39] |
| 220 – 240 | **0.02**  [0.00 ; 0.74] | **0.00**  [0.00 ; 0.01] | **0.02**  [0.00 ; 1.40] | **0.02**  [0.00 ; 0.45] | **0.05**  [0.00 ; 1.01] |
| 240 – 260 | **0.01**  [0.00 ; 0.48] | **0.00**  [0.00 ; 0.01] | **0.02**  [0.00 ; 0.44] | **0.02**  [0.00 ; 0.30] | **0.03**  [0.00 ; 0.83] |
| 260 – 280 | **0.01**  [0.00 ; 0.25] | **0.00**  [0.00 ; 0.01] | **0.01**  [0.00 ; 0.22] | **0.01**  [0.00 ; 0.20] | **0.03**  [0.00 ; 0.62] |
| 280 – 300 | **0.01**  [0.00 ; 0.22] | **0.00**  [0.00 ; 0.01] | **0.01**  [0.00 ; 0.38] | **0.01**  [0.00 ; 0.17] | **0.02**  [0.00 ; 0.61] |
| 300 – 320 | **0.01**  [0.00 ; 0.18] | **0.00**  [0.00 ; 0.01] | **0.01**  [0.00 ; 0.34] | **0.01**  [0.00 ; 0.18] | **0.02**  [0.00 ; 0.40] |

Supplementary Table S2. **Relative power contents obtained for all EGM types in the different spectral ranges**.

Percentage values are reported as medians (in bold) and 5^th^ and 95^th^ percentiles (in brackets).

| Frequency  range  (Hz) | Border | Scar | LP1 | LP2 | EP |
| --- | --- | --- | --- | --- | --- |
| 0 – 20 | **43.56**  [15.46 ; 70.20] | **67.93**  [29.52 ; 83.74] | **40.09**  [10.26 ; 59.73] | **34.14**  [7.24 ; 67.71] | **34.42**  [6.32 ; 65.22] |
| 20 – 40 | **44.46**  [12.10 ; 60.18] | **28.08**  [13.44 ; 54.13] | **43.97**  [20.66 ; 55.76] | **35.02**  [15.68 ; 58.63] | **35.50**  [11.15 ; 60.28] |
| 40 – 60 | **4.59**  [0.39 ; 25.07] | **1.09**  [0.22 ; 10.97] | **6.10**  [1.71 ; 33.78] | **9.52**  [2.48 ; 25.29] | **8.72**  [2.33 ; 31.05] |
| 60 – 80 | **0.70**  [0.06 ; 15.56] | **0.65**  [0.14 ; 5.40] | **1.80**  [0.60 ; 10.20] | **4.84**  [0.87 ; 14.24] | **3.53**  [0.56 ; 16.07] |
| 80 – 100 | **0.42**  [0.04 ; 12.60] | **0.21**  [0.05 ; 2.40] | **1.06**  [0.23 ; 4.23] | **2.38**  [0.23 ; 11.83] | **1.95**  [0.34 ; 12.85] |
| 100 – 120 | **0.29**  [0.03 ; 7.93] | **0.13**  [0.04 ; 1.20] | **0.48**  [0.06 ; 4.37] | **1.39**  [0.17 ; 8.78] | **1.19**  [0.15 ; 8.35] |
| 120 – 140 | **0.14**  [0.01 ; 5.13] | **0.09**  [0.01 ; 0.67] | **0.18**  [0.04 ; 2.20] | **0.83**  [0.08 ; 6.17] | **1.03**  [0.09 ; 5.20] |
| 140 – 160 | **0.10**  [0.02 ; 2.80] | **0.05**  [0.01 ; 0.60] | **0.15**  [0.01 ; 1.96] | **0.49**  [0.05 ; 4.67] | **0.60**  [0.08 ; 5.52] |
| 160 – 180 | **0.05**  [0.01 ; 2.27] | **0.07**  [0.01 ; 0.72] | **0.10**  [0.02 ; 3.74] | **0.45**  [0.05 ; 2.92] | **0.45**  [0.06 ; 4.16] |
| 180 – 200 | **0.04**  [0.00 ; 1.96] | **0.05**  [0.01 ; 0.45] | **0.09**  [0.02 ; 4.38] | **0.24**  [0.03 ; 2.13] | **0.37**  [0.05 ; 3.79] |
| 200 – 220 | **0.03**  [0.00 ; 1.52] | **0.05**  [0.01 ; 0.31] | **0.08**  [0.01 ; 3.95] | **0.11**  [0.02 ; 1.25] | **0.22**  [0.02 ; 2.84] |
| 220 – 240 | **0.02**  [0.00 ; 0.85] | **0.03**  [0.01 ; 0.27] | **0.06**  [0.01 ; 1.44] | **0.13**  [0.02 ; 0.84] | **0.25**  [0.04 ; 2.18] |
| 240 – 260 | **0.02**  [0.00 ; 0.56] | **0.04**  [0.00 ; 0.27] | **0.05**  [0.01 ; 0.59] | **0.11**  [0.02 ; 0.71] | **0.19**  [0.02 ; 1.87] |
| 260 – 280 | **0.01**  [0.00 ; 0.49] | **0.02**  [0.01 ; 0.30] | **0.04**  [0.00 ; 0.45] | **0.08**  [0.01 ; 0.83] | **0.18**  [0.02 ; 1.64] |
| 280 – 300 | **0.01**  [0.00 ; 0.41] | **0.03**  [0.00 ; 0.25] | **0.04**  [0.00 ; 0.61] | **0.07**  [0.01 ; 0.53] | **0.15**  [0.02 ; 1.23] |
| 300 – 320 | **0.01**  [0.00 ; 0.27] | **0.02**  [0.00 ; 0.31] | **0.04**  [0.00 ; 0.55] | **0.08**  [0.01 ; 0.97] | **0.12**  [0.02 ; 1.12] |
